# Neuropeptides and lymphocyte populations in the porcine ileum and ileocecal lymph nodes during postnatal life

**DOI:** 10.1371/journal.pone.0196458

**Published:** 2018-05-29

**Authors:** Krzysztof Wasowicz, Anna Winnicka, Jerzy Kaleczyc, Michal Zalecki, Piotr Podlasz, Zenon Pidsudko

**Affiliations:** 1 Department of Pathophysiology, Forensic Veterinary and Administration, University of Warmia and Mazury in Olsztyn, Olsztyn, Poland; 2 Division of Pathophysiology, Department of Pathology and Veterinary Diagnostic, Faculty of Veterinary Medicine, Warsaw University of Life Sciences, Warsaw, Poland; 3 Department of Animal Anatomy, Faculty of Veterinary Medicine, University of Warmia and Mazury in Olsztyn, Olsztyn, Poland; Medical University Innsbruck, AUSTRIA

## Abstract

The maturation-related changes in the concentrations of galanin (Gal), vasoactive intestinal polypeptide (VIP), substance P (SP) and somatostatin (Som), as well as in subpopulations of lymphocytes expressing antigens CD2 (lymphocytes T), CD4 (T helper), CD8 (T cytotoxic), CD21 (B lymphocytes), CD5-/CD8+ (NK cells) and TCRgamma/delta (gut mucosal/intraepitelial cells) were studied in the ileal Peyer’s patches and ileo-cecal lymph nodes in female pigs aged 3 days, 2 weeks, 4 weeks and 4 months. As regards neuropeptide concentrations statistically significant changes in the ileum and lymph nodes were found only in case of Gal and VIP. The concentrations of neuropeptides were significantly higher only in new-born animals. As regards the changes in subpopulations of lymphocytes, statistically significant changes were noticed only in 4-months old animals and were dealing only with CD2+ and TCRgamma/delta cells in the ileum as well as CD4+, CD8+, CD21+ and TCRgamma/delta in lymph nodes. The highest number of CD8+, CD21+ and TCRgamma/delta lymphocytes occurred in 4-months old animals.

## Introduction

The permeable, for the sake of absorption of nutrients, nature of the mucosa of intestines makes it a preferred gate of entry of microorganisms and needs a close monitoring by an immune system. The gastrointestinal tract-associated lymphatic tissue (GALT) in the gut consists of a diffuse population of lymphocytes and plasma cells present in the epithelium and lamina propria of mucosa, as well as of lymphatic follicles, organized regions of lymphatic tissue in the large intestine as well as in small intestine, where they are known as Peyer’s patches (PP) [1]. It collaborates with a vast collection of lymph nodes, located usually in the mesentery, filtering the lymph drained from the intestinal wall. Protective function of GALT is extremely important for normal functions and maintaining the homeostasis of the gastrointestinal tract but it is also involved in inflammatory processes, like intestinal infections, ulcerative colitis, or inflammatory bowel disease. Experimental studies on the immune system in the gut are of great significance for biomedical sciences and they need a suitable model rendering results sound for medical applications. It appears that the best model animal for studying the physiology and pathology of the gastrointestinal system is pig [2], which, as an omnivore, is closer to humans then other animal species. The studies on the (patho)physiology of the gut immune system in the pig are also important for veterinary medicine, as this species is of an extreme economical importance and disorders of the gastrointestinal tract comprise a significant share of all ailments in this species. In the porcine jejunum PP are organized as organized lymphoid follicles, but the ileum contains a continuous lymphoid follicle (lymphatic plate) extending from the distal ileum to the proximal colon [3]. The functional significance of such an organization of this follicle is unknown, but it may be speculated that it is a massive gathering of lymphoid tissue which guards the border of the small intestine (jejunum and ileum), in which the number of bacteria is moderate and the large intestine where the abundant microflora exists, containing some microorganisms which are potentially pathogenic.

Functions of the immune cells in the gut are coordinated by a vast network of regulatory substances, interleukins and chemokines, but are also modulated by the enteric nervous system, which is involved in the regulation of inflammation and immunity during pathological processes [4–6]. Many lymphatic organs receive input from cholinergic and adrenergic neurons [7]. Adrenergic and cholinergic nerve fibers release also neuropeptides being co-transmitters and neuromodulators affecting also immune cells [8]. These cells express receptors for catecholamines, somatostatin (Som), substance P (SP), vasoactive intestinal polypeptide (VIP), galanin (Gal), or neuropeptide Y (NPY) which modulate their activation, proliferation and/or immunoglobulin production [9, 10].

It is known that the gastrointestinal tract development continues after birth and the feeding is the primary stimulatory factor promoting intestinal maturation [11], affecting also changes in the enteric nervous system. Resulting contact with invading microorganisms is crucial for the development and maturation of the gut-associated immune system. In animals like pigs, the development of alimentary tract is additionally associated with an abrupt change in diet associated with weaning [12].

Taking into consideration all mentioned above facts we decided to study the changes in the innervation of Peyer’s patches of the porcine ileum and in ileocecal lymph nodes associated with the development and maturation of the gastrointestinal tract during postnatal life. The time-points selected were 3 days (newborn animals), 2 weeks (one week before weaning), 4 weeks (one week after weaning) and 4 months (mature animals). We decided to study the changes in the characteristics of GALT-associated nerve structures containing Gal, SP, Som and VIP both on the level of morphology (using immunohistochemistry and qualitative assessment) and the neuropeptides tissue concentration (assayed quantitatively with ELISA). These changes were correlated with changes in characteristic subpopulations of lymphocytes present in GALT and lymph nodes, namely lymphocytes B (CD21+), T (CD2+ including CD8+ cytotoxic and CD4+ helper cells), natural killers (CD5-/CD8+) and gut mucosal/intraepithelial (TCRgamma/delta+) lymphocytes studied at the level of morphology (immunohistochemistry and qualitative assessment) and quantitative assay (quantitative assessment with flow cytometry).

## Materials and methods

The handling of animals and all experimental procedures were in accordance with the rules of the National Ethics Commission for Animal Experimentation (Polish Ministry of Science and Higher Education). The protocol was approved by the Local Ethics Committee of the University of Warmia and Mazury in Olsztyn (Permission No. 48/2009 dated 24.06.2009) affiliated to the National Ethics Commission for Animal Experimentation (Polish Ministry of Science and Higher Education). All efforts were made to minimize animals suffering in each step of the experiment.

The study was performed on female pigs of the Large White Polish breed aged 3 days (n = 10), 2 weeks (n = 5), 4 weeks (n = 5) and 4 months (n = 5). Animals were clinically healthy. They were purchased from a commercial fattening farm.

Five 3-days old animals were used for flow cytometry study and neuropeptide assay. Animals were killed with thiopental (Thiopental, Sandoz, Austria) overdose and the abdominal cavity was opened. Ileocecal lymph nodes and part of the ileum adjacent to colon were excised and placed on ice. Excised segment of ileum was opened and the lymphatic plate was identified. For neuropeptide assays the samples of lymph node and ileum with lymphatic plate (ca. 200 mg) were excised, weighted, wrapped in Parafilm and alufoil, snap-frozen in liquid nitrogen and stored in liquid nitrogen until processed. The samples for flow cytometry were processed fresh.

Five 3-days old animals were used for immunohistochemical study. The animals were killed with thiopental (Thiopental, Sandoz, Austria) overdose and the thoracic cavity was opened. The canula was inserted into the left hearth ventricle and the animals were perfused with 4% paraformaldehyde solution in 0.1 M phosphate buffer (PB, pH 7.4). The terminal part of ileum and ileocecal lymph nodes were excised and post-fixed for 30 min. in the same fixative as used for perfusion. Subsequently, tissues were rinsed in PB for 16–18 h and then transferred to 18% sucrose solution in PB containing 0.1% of sodium azide as preservative.

In case of remaining three groups animals were deeply anesthetized with thiopental (Thiopental, Sandoz, Austria) and the abdominal cavity was opened. The studied lymph nodes and the fragment of ileum were excised and processed as for flow cytometry study and neuropeptide assay. Then the animals were killed with thiopental overdose, the thoracic cavity was opened and the animals were transcardially perfused with 4% paraformaldehyde, as described earlier. Then remaining lymph nodes and segment of ileum were excised and processed for immunohistochemistry as described earlier.

### Flow cytometry and neuropeptide assays

Flow cytometry analysis was performed according to method of Kaleczyc et al. (2010) [13]. For flow cytometry studies the lymph node was cut by half with scalpel blade, the half of the lymph node was put flat on the cooled Petri dish with the cut surface facing up and the stroma of the lymph node was repeatedly chopped with a scalpel blade. Then the tissue was placed in 1,5 ml of ice-cold PBS (phosphate-buffered saline, pH 7.4) in 2 ml Eppendorf tubes. The fragment of the ileum was opened, rinsed with PBS, the lymphatic plate was identified and the fragment of the ileum placed with a mucosal surface facing up. The mucosa was scraped and chopped simultaneously with a scalpel blade. The scraped mucosa was placed in 1,5 ml of ice-cold PBS in 2 ml Eppendorf tubes. The chopped tissues were shaken for 1 min. in PBS, the tissue fragments were allowed to sedimentate for 2 min. and the suspensions were removed. The “extraction” was repeated. The pooled suspensions were filtered through a polyester wool using 2 ml disposable syringes as filtration columns. Then the concentrations of lymphocytes in suspensions were measured in haemocytometer. Viability of lymphocytes was assessed with 0.4% Trypan Blue staining. Equal volumes of cell suspension and 0,4% Trypan Blue mixed, put into haemocytometer and left for 2 mins. Viable cells were colorless and nonviable ones were colored blue.

For flow cytometry assays the suspensions were prepared containing 10^6^ lymphocytes in 50 μl of PBS. 50 μl of suspension were placed in seven 2 ml Eppendorf tubes marked A-G. Appropriate antibodies (primary and secondary) were added to tubes and suspensions were incubated for 30 min. at room temperature (RT). The data of primary antibodies are shown in Table 1.

**Table 1:** Data of primary antibodies.

No.	Antigen	Antibody	Cat. No.
P1	CD2	IgG2a	MSA4
P2	CD4	IgG2b	74-12-4
P3	CD5	IgG1	PG114A
P4	CD8	IgG2a	76-2-11
P5	CD21	IgG1	BB6-11C9
P6	TCRγδ	IgG1	86D

All antibodies were mouse monoclonal antibodies purchased from VMRD Inc., USA

The data of secondary antibodies are shown in Table 2.

**Table 2:** Data of secondary antibodies.

Symbol	Antigen or ligand	Marker	Cat. No.
S1	anti-Mouse IgG1	PE	550083
S2	anti-Mouse IgG2a	FITC	553390
S3	anti-Mouse IgG2b	Biotin	550333
S-PE	biotin	PE	554061

Secondary antibodies and streptavidin conjugated with phycoerithrine (PE) were purchased from Pharmingen. All antibodies were anti-mouse antibodies.

The sequence of antibodies used is shown in Table 3.

**Table 3:** Procedure used for staining CD antigens in lymphocyte suspensions.

A	B	C	D	E	F	G
negative control	Background control	Background control	CD2/CD21	CD4/CD8	CD5/CD8	TCRγδ/CD8
lymphocyte suspension	lymphocyte suspension	lymphocyte suspension	lymphocyte suspension	lymphocyte suspension	lymphocyte suspension	lymphocyte suspension
	1 μl S1	1 μl S2	1 μl P1 + 1 μl S2	1 μl P2 + 1 μl S3 + 1 ml S-PE	1 μl P3 + 1 μl S1	1 μl P6 + 1 μl S1
Washing	Washing	washing	Washing	washing	washing	washing
FA	FA	FA	1 μl P5 + 1 μl S1	1 μl P4 + 1 μl S3	1 μl P4 + 1 μl S3	1 μl P4 + 1 μl S3
END	END	END	Washing	washing	washing	washing
			FA	FA	FA	FA
			END	END	END	END

2 ml of PBS were added to each tube, mixed gently and centrifuged 5 min at 1200 rpm. The supernatants were removed and the sediments were dispersed with gentle pipetting. 300 μl of 1% formaldehyde (FA) was added to tubes A, B and C, the suspension was gently mixed and set aside. Appropriate primary and secondary antibodies were added to tubes D-G and tubes were incubated for 30 min. at RT. Then 2 ml of PBS was added, tubes were gently mixed centrifuged for 5 min. at 1200 rmp. The majority of supernatants were removed, the sediments were resuspended in the remaining supernatant and 300 μl of 1% formaldehyde (FA) was added with gentle mixing. Lymphocyte suspensions were analyzed in a flow cytometer (Beckton Dickinson FACScalibur) and the data were analyzed with Cell Quest software (Beckton Dickinson). Data were statistically analyzed with a GraphPad Prism Software (GraphPad Software) using ANOVA and Student-Newman-Tukey multiple comparison post-test. Differences were regarded as statistically significant at p<0.01.

Neuropeptide extraction was performed according to the modified method of Brill et al. [14]. For neuropeptide assays samples were taken out of the liquid nitrogen, homogenized with a high-speed homogenizer (UltraTurrax, Germany) in 0.5 M acetic acid at 4°C and placed in boiling water bath for 10 min. After cooling on ice the homogenates were centrifuged for 20 min. at 10000g, the clear supernatants were collected and the pellets were re-extracted twice. The supernatants were pooled and lyophylized. The dried extracts were dissolved in 2 ml of water and stored frozen at –70°C. Tissue concentrations of VIP, Som, SP and Gal were determined with ELISA tests using commercial kits (Peninsula Laboratories, USA) according to the manufacturer’s instructions. Data of the ELISA kits are listed in Table 4.

**Table 4:** Enzyme immunoassay kits used (Peninsula Laboratories Inc.).

Substance	Cat. No.	Lot No.
VIP	S-1183 (EIAH-7161)	016535
Gal	S-1210 (EIAH-7100)	016537
Som	S-1179 (EIAH-8001)	016538
SP	S-1180 (EIAH-7451)	016536

ELISA plates were read with a Dynex MRX (Dynex Technologies, USA) immunoplate reader equipped with a 450 nm filter. Ten-point standard curve was prepared and absorbances were converted to peptide concentrations. The results were re-calculated for 1 g of fresh tissue. Data were statistically analyzed with a GraphPad Prism Software (GraphPad Software) using ANOVA and Student-Newman-Tukey multiple comparison post-test. Differences were regarded as statistically significant at p<0.01.

### Immunohistochemistry

For stainings of CD2, CD21, CD4, CD8, TCRgamma/delta antigens, as well as of Gal the tissues were taken out of liquid nitrogen, placed in a cryostat (temperature -22°C) overnight to equilibrate, put on cryostat holders covered with OTC compound and allowed to freeze. Then, 20 μm-thick sections were cut and put on a chrome alum-gelatin-coated slides. Then slides were put into ice-cold methanol for 5 min and transferred to phosphate-buffered saline (PBS) at room temperature to rehydrate. Rehydrated slides were processed for indirect immunofluorescence.

Stainings for CD2, CD21 antigens, as well as for VIP, Som and SP were done also in tissues fixed with 4% paraformaldehyde. 20 μm-thick frozen sections were cut with a cryostat, put on chrome alum-gelatin-coated microscope slides, dried for 20 min., put into storage boxes and stored desiccated at -70°C until needed. Slides were taken out of boxes, allowed to dry for 20 min, and put into PBS to rehydrate. Rehydrated slides were used for indirect immunofluorescence.

The data of primary antibodies against lymphocyte antigens and neuropeptides, as well as the data of secondary antibodies are listed in Table 5.

**Table 5:** Data of primary and secondary antisera used for immunofluorescence stainings.

Antigen	Species	Code	Dilution	Supplier
PRIMARY ANTIBODIES				
VIP	Rabbit	AB22736	1:5000	Abcam
Gal	Rabbit	Rin-7153	1:2000	Peninsula
Som	Rabbit	LS-C39186	1:6000	LifeSpan Biosciences
SP	Rabbit	LS-C42165	1:1000	LifeSpan Biosciences
CD2	Mouse	MSA4	1:5000	VMRD
CD4	Mouse	74-12-4	1:1000	VMRD
CD8	Mouse	76-2-11	1:500	VMRD
CD21	Mouse	BB6-11C9	1:500	VMRD
TCR_gamma/delta_	Mouse	86D	1:500	VMRD
SECONDARY ANTIBODIES				
Alexa Fluor® 488 anti-mouse IgG	Goat	A-11001	1:500	Invitrogen
Alexa Fluor® 546 anti-rabbit IgG	Goat	A-11010	1:500	Invitrogen

The slides were rinsed with phosphate buffered saline (PBS, pH 7.4), preincubated with a blocking mixture containing 0.1% Triton X-100 and 10% normal goat serum in PBS (1 h, RT), and incubated overnight (18-20h) at RT in a humid chamber with the primary antisera at proper concentrations in a blocking solution. After incubation with primary antibody, the slides were washed in PBS and incubated with secondary antisera diluted 1:500 in PBST and 10% normal goat serum for 1 hour at room temperature. After that the slides were washed with PBS and mounted with glycerol and PBS (1:1).

All specimens were examined with a Zeiss LSM 710 confocal microscopy system. The obtained images were assessed qualitatively for differences in the distribution and density of immunostained structures independently by three researchers.

## Results

### Ileum

#### Neuropeptides immunohistochemistry

No age-related differences regarding the number and distribution of the nerve fibers containing the studied neuropeptides were found.

**Fig 1:**
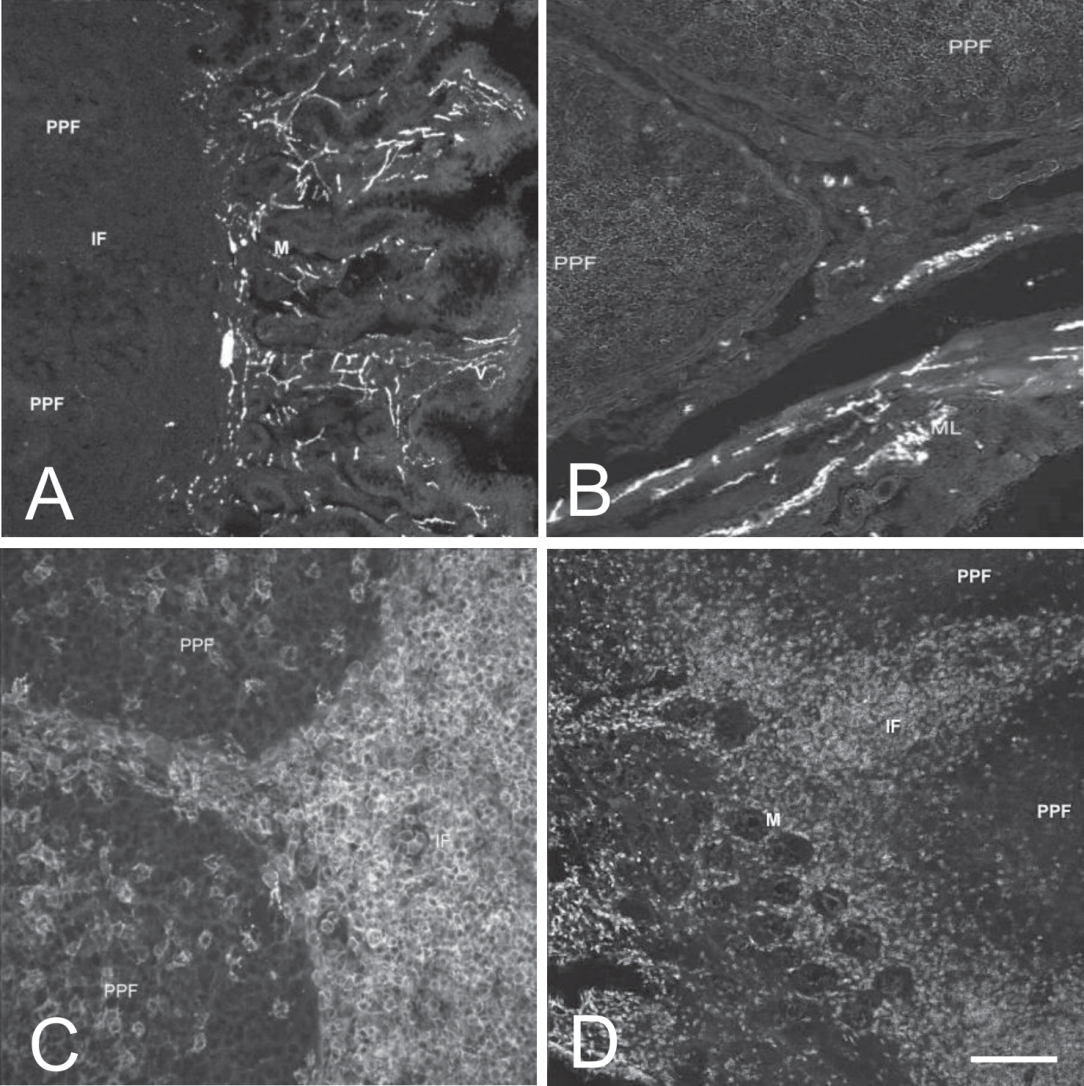
A–Gal-positive nerve fibers in the mucosal membrane of the ileum of 4 weeks-old animals. B–VIP-positive nerve fibers in the muscular membrane of the ileum of 4 weeks-old animals. C–CD2+ lymphocytes in the mucosal membrane of the ileum of 4 weeks-old animals. D–CD8+ lymphocytes in the mucosal membrane of the ileum of 4 weeks-old animals. Abbreviations: PFF–Peyer’s patch follicle, IF–interfollicular region, M–mucosa, ML–muscular layer. Bar: Fig A, D– 100 μm, B– 50 μm, C– 25 μm.

Gal-positive nerve fibers were numerous in the mucosa (Fig 1A), submucosa and muscular membrane. The fibers were absent in lymphatic follicles, single fibers were present around follicles and fibers were numerous in the interfollicular area.

VIP-positive nerve fibers were very numerous in the mucosa and submucosa. They were less numerous in the muscular membrane (Fig 1B). Nerve fibers were absent in the lymphatic follicles, single fibers were seen in the follicles periphery and numerous VIP-positive nerve fibers were present in the interfollicular area.

In all experimental groups Som-positive nerve fibers were moderately numerous in the mucosa and submucosa (Data not shown). These fibers were scarce in the muscular membrane. No Som-positive nerve fibers were seen in the follicles and in their periphery. Moderately numerous Som-positive nerve fibers were seen in the interfollicular area.

SP-positive nerve fibers were numerous in the mucosa and submucosa, while their number in the muscular layer was even larger (Data not shown). No SP-positive nerve fibers were present in the lymphatic follicles, single fibers were visible in their periphery and moderately numerous SP-positive nerve fibers were seen in the interfollicular regions.

#### Neuropeptide ELISA assays

Measurements of tissue concentrations of the studied neuropeptides in the ileal wall showed that statistically significant differences between experimental groups regarded only Gal and VIP. The highest concentrations of these neuropeptides were seen in 3-days old animals. In animals aged 2 weeks, 4 weeks and 4 months concentrations were lower. No statistically significant differences were seen between animals aged 2 weeks, 4 weeks and 4 months. Statistically significant differences were seen between 3-days old animals and other groups in case of VIP. In case of Gal statistically significant differences were found between 3day old animals and animals aged 4 weeks and 4 months. Results in animals aged 2 weeks were statistically insignificant. Data are shown in Table 6.

**Table 6:** Concentrations of the studied neuropeptides in the ileum shown as ng/g wet weight.

	a	b	c	d
	3 days	2 weeks	4 weeks	4 months
Gal	27.15+3.74cd	19.16+4.2	11.36+1.2a	14.39+0.73a
VIP	20.80+4.1bcd	2.72+0.45a	3.00+0.58a	3.92+0.52a
Som	3.15+1.65	1.09+0.24	1.15+0.18	1.33+0.20
SP	2.96+0.67	2.49+0.36	2.00+0.20	1.84+0.38

Data presented as mean +SEM. Letters indicate groups to which particular result is statistically significantly different.

#### Lymphocyte subpopulations immunohistochemistry

CD2+ lymphocytes were scarce in the lymphatic follicles, but very numerous in the interfollicular region (Fig 1C). They were also present in the mucosa and mucosal epithelium. Apparently, their number was increasing with age in the interfollicular area, and decreasing in the epithelium.

CD4+ lymphocytes were scarce in the follicles (in the center their number was higher than in the periphery) and moderately numerous in the interfollicular area. They sporadically occurred in the mucosa (Data not shown). No age-related differences between groups were seen.

CD8+ lymphocytes were scarce in the follicles and moderately numerous in the interfollicular area. CD8+ lymphocytes were numerous in the mucosa and mucosal epithelium (Fig 1D). No age-related differences were seen between experimental groups.

**Fig 2:**
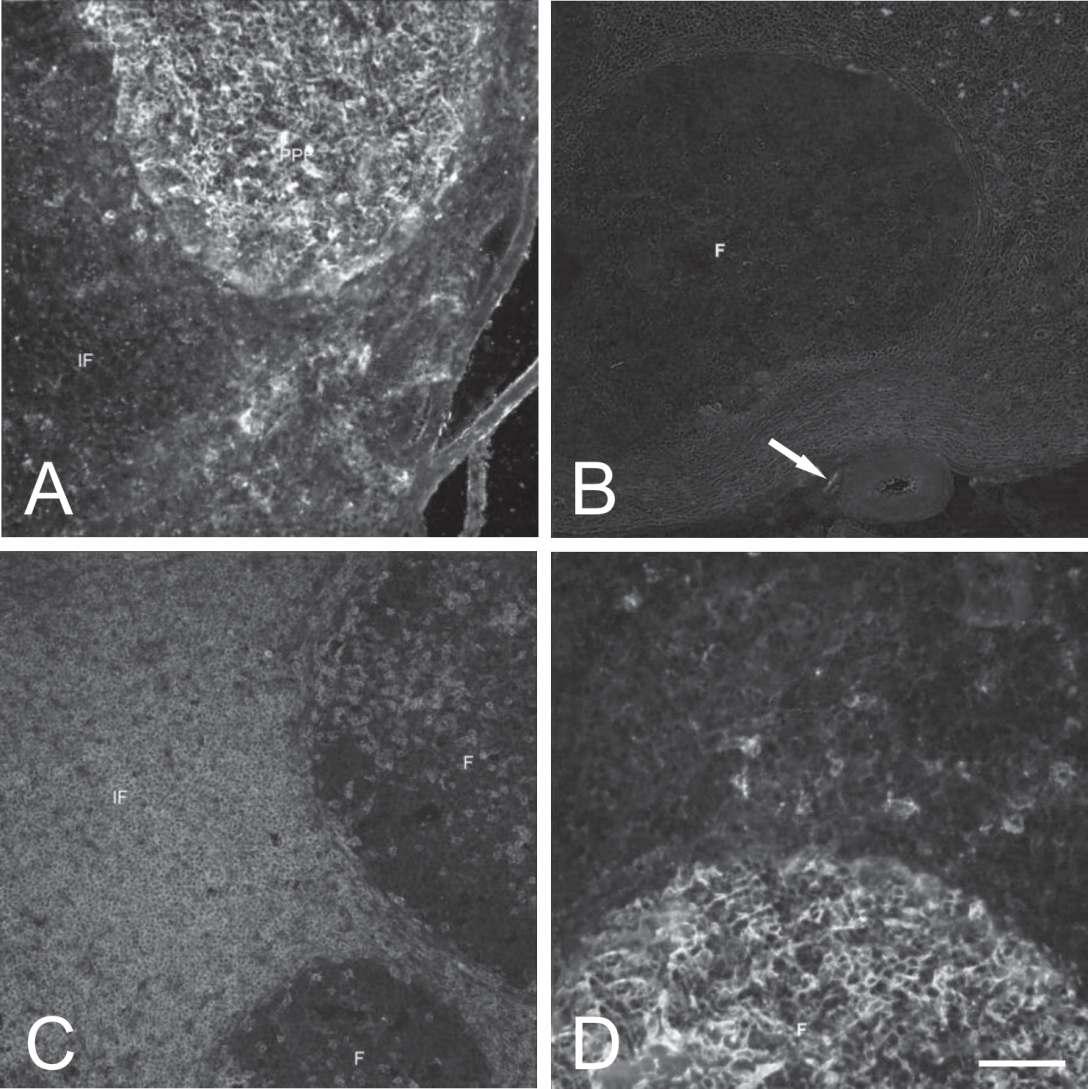
A–CD21+ lymphocytes in the mucosal membrane of the ileum of 4 weeks-old animals. B–Single SP-positive nerve fiber (arrow) in the vicinity of the blood vessle in the lymph node of 4 weeks-old animal. C–CD2+ lymphocytes in the lymph node of 4 weeks-old animal. D–CD21+ lymphocytes in the lymph node of 4 weeks-old animal. Abbreviations: F–lymphatic follicle, IF–interfollicular region, M. Bar: Fig A, B, C– 50 μm, D– 25 μm.

CD21+ lymphocytes were very numerous in lymphatic follicles and only single occurred in other regions of the intestinal wall (Fig 2A). No age-related differences were noticed between experimental groups.

TCRgamma/delta+ lymphocytes were absent in the lymphatic follicles and only single cells were seen in the interfollicular area and in the mucosa (Data not shown). No age-related differences were seen between experimental groups.

#### Lymphocyte subpopulations flow cytometry assays

Flow cytometry detected statistically significant differences in case of CD2+, CD5-/CD8+ (NK) and TCRgamma/delta+ lymphocyte subpopulations. The numbers of these lymphocytes were highest in 4-months old animals, while in remaining, younger animals they were much lower. Statistically significant differences were detected between 4-months old animals and remaining groups. Data are shown in Table 7.

**Table 7:** Percentages of the studied populations of lymphocytes in the ileum.

	a	b	c	d
	3 days	2 weeks	4 weeks	4 months
CD21	38.36+13.53	67.23+4.01	51.08+6.69	64.18+8.24
CD2	11.12+2.65d	10.32+1.07d	11.27+0.95d	32.81+3.96abc
CD4	22.74+5.10	11.19+0.89	12.40+2.07	19.38+3.49
CD8	26.48+10.76	3.59+0.47	3.72+1.09	18.02+2.15
NK	7.38+5.24	0.56+0.21	0.64+0.28	0.67+0.12
TCRgd	5.63+1.46d	1.97+0.30d	4.22+1.45d	22.25+8.32abc

Data presented as mean+SEM.

Letters indicate groups to which particular result is statistically significantly different.

### Lymph nodes

#### Neuropeptides immunohistochemistry

Studies on the number and distribution of nerve fibers containing the studied neuropeptides revealed no age-related differences.

No Gal-positive nerve fibers were seen in the stroma of the lymph nodes. They occurred sporadically in the node hilus and in vicinity of blood vessels. Such fibers were numerous in the connective tissue surrounding the lymph nodes (Data not shown).

No VIP-positive nerve fibers were seen in the stroma of the lymph nodes. They occurred sporadically in the node hilus and in vicinity of blood vessels. Such fibers were also scarce in the connective tissue surrounding the lymph nodes (Data not shown).

No Som-positive nerve fibers were seen in the lymph nodes.

No SP-positive nerve fibers were seen in the stroma of the lymph nodes. They occurred sporadically in the node hilus and in vicinity of blood vessels (Fig 2B). Such fibers were also scarce in the connective tissue surrounding the lymph nodes.

#### Neuropeptide ELISA assays

Measurements of tissue concentrations of the studied neuropeptides in the lymph nodes showed that statistically significant differences between experimental groups regarded only Gal and VIP. The highest concentrations of Gal was seen in 4-weeks old animals and this difference was statistically significant to other groups. As regards VIP the concentration was highest in 3-weeks old animals and this difference was statistically significant to other groups. No other statistically significant differences were detected. Data are shown in Table 8.

**Table 8:** Concentrations of the studied neuropeptides in lymph nodes shown as ng/g wet weight.

	a	b	c	d
	3 days	2 weeks	4 weeks	4 months
Gal	12.29+4.06c	10.94+1.62c	22.48+2.37abd	12.04+1.84c
VIP	4.81+1.39cbd	1.26+0.29a	1.33+0.12a	1.29+0.35a
Som	1.30+0.48	0.79+0.16	1.56+0.49	1.03+0.13
SP	3.20+1.10	3.18+0.46	3.02+0.47	5.29+0.46

Data presented as mean+SEM.

Letters indicate groups to which particular result is statistically significantly different.

#### Lymphocyte subpopulations immunohistochemistry

CD2+ lymphocytes were scarce in the follicles and very numerous in the interfollicular area (Fig 2C). Apparently, their number was increasing with age in the interfollicular area.

CD4+ lymphocytes were scarce in the follicles and moderately numerous in the interfollicular area (Data not shown). No age-related differences between experimental groups were seen.

CD8+ lymphocytes were seen only sporadically in the lymphatic follicles, but they were numerous in the interfollicular area (Data not shown). There were no visible differences between groups related to age of animals.

CD21+ lymphocytes were very numerous in the follicles (Fig 2D) and only single cell were present in remaining areas of the lymph nodes. There were no age-related differences between experimental groups.

TCRgamma/delta+ lymphocytes were absent in the lymphatic follicles. In other regions of lymph nodes they were seen only occasionally.

#### Lymphocyte subpopulations flow cytometry assays

Flow cytometry revealed statistically significant differences regarding the number of CD4+ lymphocytes (apparent rise in 2-weeks old animals), as well as CD8+ and TCRgamma/delta+ lymphocytes (the apparent rise in 4-months old animals). Data are shown in Table 9.

**Table 9:** Percentages of the studied populations of lymphocytes in lymph nodes.

	a	b	c	d
	3 days	2 weeks	4 weeks	4 months
CD21	20.64+6.07d	32.34+1.93	39.47+3.19	52.73+8.65a
CD2	34.57+7.05	56.3+1.77	45.19+4.29	50.05+9.85
CD4	36.25+4.89b	59.36+5.96acd	36.57+2.69b	30.78+5.95b
CD8	10.31+1.54d	11.48+1.17d	12.49+1.78d	24.80+1.89abc
NK	0.58+0.21	0.87+0.1	1.10+0.20	0.56+0.08
TCRgd	1.83+0.46d	2.04+0.26d	3.04+0.27d	26.15+10.36abc

Data presented as mean+SEM.

Letters indicate groups to which particular result is statistically significantly different.

## Discussion

Interestingly, although it was in accordance with earlier studies [15], no nerve fibers containing the studied neuropeptides were seen inside the lymphatic follicles of the gut and lymph nodes. However, disregarding lymphocytes B (CD21+), which occurred in the great number in the follicles, the remaining lymphocyte subpopulations were more numerous in the interfollicular area than in the follicles. However, the neuropeptides are neurotransmitters and neuromodulators showing, so called, “volume transmission” [16, 17], what means that they do not need to be released into the synaptic cleft but can diffuse in the tissue over long distances and stimulate receptors located on cells making no direct contact with nerve fibers, Lack of a clear spatial relationship between nerve fibers and lymphocytes doesn’t exclude modulatory effect of the studied neuropeptides on the growth, maturation and activity of immune system cells.

No apparent differences in the number and distribution of nerve fibers containing Gal, VIP, Som and SP between animals of different age, ranging from 3 days to 4 months were seen. It may be that the differences were subtle enough not to be easily detectable visually and detecting them would need using morphometric techniques. However, quantitative assays revealed that tissue concentrations of Gal and VIP are much higher in newborn animals, than in older groups. Since in 2-weeks old, 4-weeks old and 4-months old animals the levels of these neuropeptides are more, or less equal, it seems that the two neuropeptides are involved in the processes of adaptation of alimentary tract to contact with external environment. Gal in pig is a neuropeptide whose expression in nerve cells is up-regulated by many noxious stimuli, including axotomy and inflammation [18]. It is possible that in newborn animals the contact of the alimentary tract with the external environment, namely food containing toxins and microbes, poses a kind of stress, evoking even a sub-clinical inflammation, making adaptive changes necessary [12]. This hypothesis is somehow backed by the fact that another neuropeptide, whose concentration was raised in newborn piglets, is VIP. This neuropeptide is known to be a potent anti-inflammatory factor involved in the inflammation descent [19–21]. The question arises, why clear changes in Gal and VIP tissue concentrations are not reflected by morphological changes in the number and distribution of nerve fibers and neuronal somata containing these neuropeptides. However, it must be kept in mind that quantitative assays measure not only content of neuropeptides located in the nerve structures, but also measure a fraction already released from nerve terminals into the tissue in a kind of “snapshot image”.

The changes in the lymphocyte subpopulations in the ileum are also far from dramatic. The only sub-populations in which quantitative changes were found to be statistically significant in the ileum at the level of flow cytometry were CD2+ and TCRgamma/delta lymphocytes. However, unlike in case of Gal- and VIP-positive nerve structures, the changes in the number of CD2+ lymphocytes were seen both on the level of morphology and quantitative analysis by flow cytometry. It was found that the number of CD2+ lymphocytes was rising with age in the interfollicular area. A similar phenomenon was found in flow cytometry assays, which showed the highest percentage of CD2+ lymphocytes in 4-months old animals. CD2 antigen is a molecule being commonly regarded as a marker of T lymphocytes, regardless their other antigens. Interestingly, no statistically significant changes in the percentage of CD4+ and CD8+ lymphocytes, being very important sub-classes of T cells, were found with flow cytometry. The rise in the number of CD2+ cells can be partially attributed to the rise in TCRgamma/delta subpopulation, since these lymphocytes were more abundant in 4-months old animals. However, the rise should be also caused by changes in some other lymphocyte subpopulation. The obvious candidate is a sub-population of CD8+ lymphocytes. Although the rise was found to be statistically insignificant, the increase of the mean value from above 3% in 2- and 4-weeks old animals to 18% in 4-months old animals is striking. It is noteworthy to mention that also in 3-days old animals the number of CD8+ lymphocytes was over 26%. Finding these differences as statistically insignificant doesn’t mean there are no differences between experimental groups.

The situation with the changes in neuropeptide concentrations in ileocecal lymph nodes is similar to that in the ileum. The only differences regard concentrations of Gal and VIP and the changes are similarly pronounced as in the ileum. It suggests that processes occurring in the alimentary tract affect to the similar extent the lymph nodes filtering the lymph out-flowing from the gut. It may suggest that the main stressors affecting the maturating alimentary tract are infectious agents, but it must be kept in mind that the immune system is alerted also by various soluble antigens and VIP is involved in the development of so called “oral tolerance” [22] taking place in postnatal period.

However, as regards changes in the lymphocyte subpopulations situation in lymph nodes is more complicated. No statistically significant changes were found in case of CD2+ lymphocytes and most pronounced differences occurred in 4-months old animals in case of CD8+ and TCRgamma/delta lymphocyte subpopulations. It proves that the processes affecting the immune system at the level of the wall of the alimentary tract and intestinal lymph nodes run somehow independently and according to different schemes. The only change consistent with the changes occurring in the ileum is the tremendous rise in the number of TCRgamma/delta lymphocytes in 4-months old animals (not visible at the level of total T lymphocyte population—CD2+ cells).

Is there any correlation between changes in the Gal and VIP content and changes in the lymphocyte subpopulations? Apparently not. The highest levels of the neuropeptides were found in newborn animals and the most pronounced changes in lymphocyte subpopulations were found in 4-months old animals. This time lap suggests no direct coupling between neuropeptide level and changes in lymphocyte subpopulations. However, there is possibility that the link still exists, albeit masked by its complex mechanism. The influence of Gal on the lymphocytes is not thoroughly studied, however, it was found that this neuropeptide has a potent anti-proliferative influence on, at least, certain lymphocyte subpopulation [23]. The high level of this neuropeptide in the alimentary tract could lead to a drop in the size of certain lymphocyte subpopulations. However, the simultaneous increase of the concentration of VIP, the neuropeptide showing stimulatory and anti-apoptotic effect on lymphocytes [24–26], may counteract this effect. This may be the case of balanced response of the alimentary tract, enteric nervous system and immune system to the challenge imposed by alimentary factors.

Our results failed to detect a direct link between changes in the neuropeptide distribution and concentrations as well as in changes in the studied lymphocyte sub-populations during maturation of porcine alimentary tract. However, it still cannot be excluded and, most probably, this link exists. However, more studies are necessary to elucidate this problem.
